# First person – Kristin Ates

**DOI:** 10.1242/dmm.044818

**Published:** 2020-05-26

## Abstract

First Person is a series of interviews with the first authors of a selection of papers published in Disease Models & Mechanisms, helping early-career researchers promote themselves alongside their papers. Kristin Ates is first author on ‘ Deficiency in the endocytic adaptor proteins PHETA1/2 impairs renal and craniofacial development’, published in DMM. Kristin is an MD/PhD student in the lab of Y. Albert Pan at Augusta University, Augusta, GA, USA, investigating the underlying pathogenesis of endosomal and lysosomal diseases.


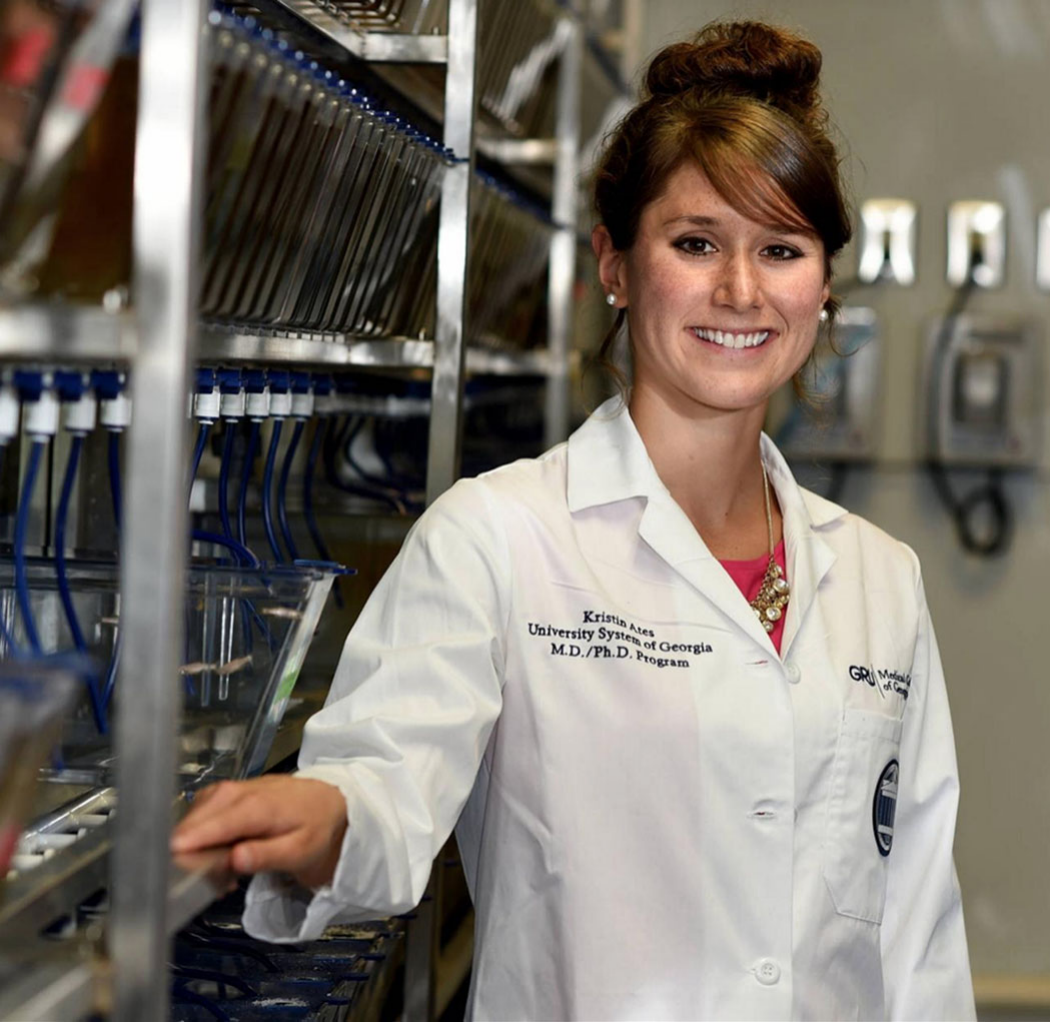


**Kristin Ates**

**How would you explain the main findings of your paper to non-scientific family and friends?**

A critical barrier in the treatment of endosomal and lysosomal diseases is the lack of understanding of the *in vivo* functions of the putative causative genes. Examples of such endosomal diseases include Charcot-Marie-Tooth disease and autosomal dominant kidney disease. We addressed this by investigating a key pair of endocytic adaptor proteins, PH domain-containing endocytic trafficking adaptor 1 and 2 (PHETA1/2), which interact with the protein product of *OCRL*, the causative gene for Lowe syndrome. We conducted the first study of PHETA1/2 *in vivo*, by creating a zebrafish mutant model. We found that impairment of both zebrafish *pheta1* and *pheta2* disrupted endocytosis and craniofacial development. These findings were consistent with the clinical presentation of a patient with a *de novo* arginine (R) to cysteine (C) variant (R6C) of PHETA1. Together, these results provide insights into the *in vivo* roles of PHETA1/2 and suggest that the R6C variant is contributory to the pathogenesis of disease in the patient.

**What are the potential implications of these results for your field of research?**

The regulation of endocytic trafficking is essential for the development and function of an organism. By identifying novel roles of PHETA1/2, particularly in endocytosis and craniofacial development, we have advanced the field's understanding of the importance of endocytic adaptor proteins in the pathogenesis of human disease. The continued investigation of endocytic adaptor proteins may soon reveal therapeutic targets for endosomal and lysosomal diseases, many of which have no known cure at this time.

**What are the main advantages and drawbacks of the model system you have used as it relates to the disease you are investigating?**

Zebrafish are highly genetically tractable, and approximately 70% of the human genes correspond with at least one orthog in the zebrafish genome, making this an ideal model to explore putative human disease genes, particularly PHETA1/2. Additionally, zebrafish have rapid *ex utero* development and generate large numbers of embryos, which allowed us to rapidly analyze the effects of the *pheta1/2* genetic mutations. The relationships between zebrafish lower-jaw elements and human jaw anatomy made this an invaluable model to further understand craniofacial development. However, one primary drawback of utilizing zebrafish models is that they are not as closely related to humans as other vertebrate models are, such as mouse.

**What has surprised you the most while conducting your research?**

We were not expecting to discover a novel role of PHETA1/2 in craniofacial morphogenesis. In the *pheta2^−/−^* and double mutant (*dKO*) animals, we observed features indicative of abnormal chondrocyte differentiation, including abnormal chondrocyte morphology, reduced ceratohyal ossification, changes in marker gene expression and altered extracellular matrix composition (i.e. increased type II collagen).

**Describe what you think is the most significant challenge impacting your research at this time and how will this be addressed over the next 10 years?**

One major challenge impacting our research at this time is the fact that we only have information from one human patient in regards to the clinical manifestations associated with a *de novo* PHETA1 mutation. It is important to optimize collaboration with clinical physicians to identify additional patients carrying deleterious PHETA1 mutation, which will help us to clarify which phenotypes are more closely associated with PHETA1 deficiency in humans. The National Institutes of Health's Undiagnosed Diseases Program has done an excellent job in bridging this gap between clinicians and scientists. Continued advancement of genomic sequencing and educating the general population about undiagnosed diseases will help us tackle this problem over the next 10 years.

**Figure:**
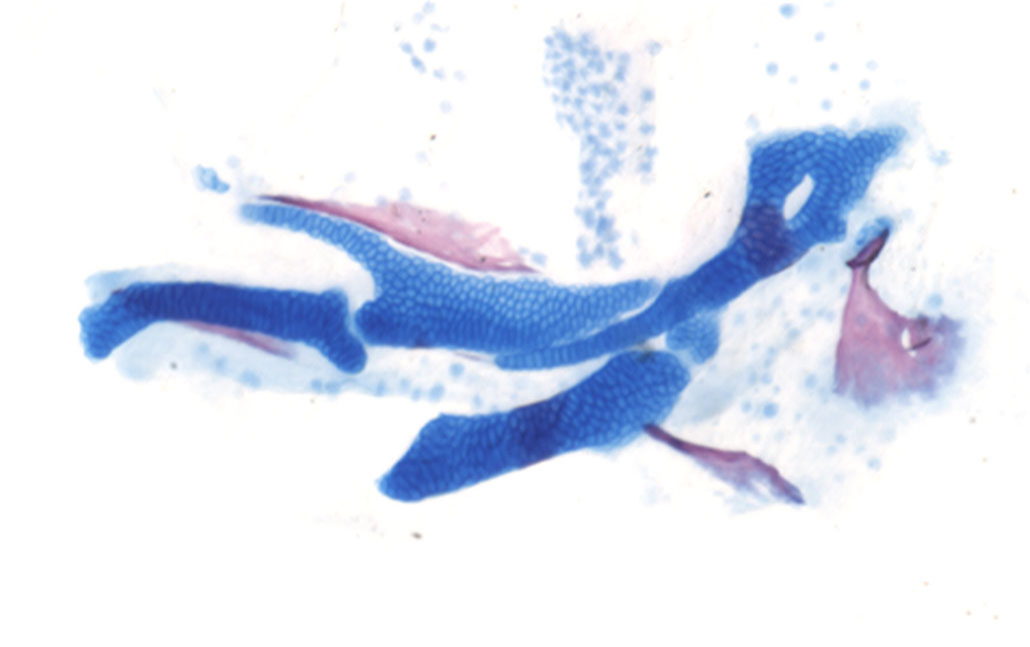
**Flat-mount preparation of the zebrafish lower jaw at 6 days post-fertilization, utilizing Alcian Blue and Alizarin Red to stain bone and cartilage, respectively.**

“[…] exposure to multiple fields of medicine and research early in the career of a scientist is invaluable.”

**What changes do you think could improve the professional lives of early-career scientists?**

I believe exposure to multiple fields of medicine and research early in the career of a scientist is invaluable. Not only does it widen one's perspective, but it also promotes a greater appreciation for other specialties. Furthermore, encouraging stronger collaboration between medical students and graduate students will enable better collaboration between future physicians and scientists. This will help solidify the common goals early in our careers, which is ultimately advancing patient care and quality of life.

**What's next for you?**

As an MD/PhD student, I'm currently pursuing a career as a physician-scientist, and plan to graduate with both MD and PhD degrees in spring 2021. My passion is advocating for patients with rare and undiagnosed diseases, and I hope to continue to do so by optimizing the collaboration between clinicians and basic scientists in my future specialty.

**What advice do you have for underrepresented students in medicine and/or basic science research?**

Be your strongest advocate, while being diligent in your work and gracious to others. I was born with bilateral sensorineural hearing loss, for which I have a cochlear implant. With the help of family, clinicians, audiologists and therapists, I learned to adapt and excel in multiple aspects of everyday life. Now, as an MD/PhD student, one of the most invaluable traits I have is the ability to personally relate with many of my patients. I am truly excited to continue down my career path as a physician-scientist and would not be where I am today without the help of many excellent mentors along the way.
